# An Apparent Diffusion Coefficient-Based Machine Learning Model Can Improve Prostate Cancer Detection in the Grey Area of the Prostate Imaging Reporting and Data System Category 3: A Single-Centre Experience

**DOI:** 10.3390/cancers15133438

**Published:** 2023-06-30

**Authors:** Caterina Gaudiano, Margherita Mottola, Lorenzo Bianchi, Beniamino Corcioni, Lorenzo Braccischi, Makoto Taninokuchi Tomassoni, Arrigo Cattabriga, Maria Adriana Cocozza, Francesca Giunchi, Riccardo Schiavina, Stefano Fanti, Michelangelo Fiorentino, Eugenio Brunocilla, Cristina Mosconi, Alessandro Bevilacqua

**Affiliations:** 1 Department of Radiology, IRCCS Azienda Ospedaliero-Universitaria di Bologna, 40138 Bologna, Italy; caterina.gaudiano@aosp.bo.it (C.G.); margherita.mottola@unibo.it (M.M.); beniamino.corcioni@aosp.bo.it (B.C.); cristina.mosconi3@unibo.it (C.M.); 2 Department of Medical and Surgical Sciences (DIMEC), University of Bologna, 40138 Bologna, Italy; lorenzo.braccischi@studio.unibo.it (L.B.); makoto.taninokuchi@studio.unibo.it (M.T.T.); arrigo.cattabriga@studio.unibo.it (A.C.); mariaadriana.cocozza@studio.unibo.it (M.A.C.); riccardo.schiavina3@unibo.it (R.S.); s.fanti@unibo.it (S.F.); michelangelo.fiorentino@unibo.it (M.F.); eugenio.brunocilla@unibo.it (E.B.); 3 Division of Urology, IRCCS Azienda Ospedaliero-Universitaria di Bologna, 40138 Bologna, Italy; lorenzo.bianchi13@unibo.it; 4 Department of Pathology, IRCCS Azienda Ospedaliero-Universitaria di Bologna, 40138 Bologna, Italy; francesca.giunchi@aosp.bo.it; 5 Department of Nuclear Medicine, IRCCS Azienda Ospedaliero-Universitaria di Bologna, 40138 Bologna, Italy; 6 Department of Computer Science and Engineering (DISI), University of Bologna, 40126 Bologna, Italy

**Keywords:** prostate cancer, machine learning, PI-RADS 3 lesions, prediction models, magnetic resonance imaging

## Abstract

**Simple Summary:**

Multiparametric Magnetic Resonance Imaging (mpMRI) interpretation and reporting is based on the more recent version 2.1 of the Prostate Imaging-Reporting and Data System (PI-RADS), revised in 2019, indicating the probability of clinically significant Prostate Cancer (csPCa) on a 5-point scale, which should be confirmed through trans-rectal ultrasound (TRUS) fusion-targeted biopsy. Among PI-RADS categories, PI-RADS 3 lesions represent a highly “equivocal” result, with a non-negligible probability of PCa, or even csPCa. This study exploits machine learning methods in order to investigate the role of mpMRI as a stand-alone tool for early and non-invasive detection of PCa in a selected cohort of PI-RADS 3 lesions, by means of a radiomic analysis of Apparent Diffusion Coefficient sequences. Differently from what reported in the current literature, the methodology adopted has bounded the possibility of overoptimistic predictive performance, also improving the state-of-art by achieving a positive predictive value of 80%, with specificity = 76% and sensitivity = 78%.

**Abstract:**

The Prostate Imaging and Reporting Data System (PI-RADS) has a key role in the management of prostate cancer (PCa). However, the clinical interpretation of PI-RADS 3 score lesions may be challenging and misleading, thus postponing PCa diagnosis to biopsy outcome. Multiparametric magnetic resonance imaging (mpMRI) radiomic analysis may represent a stand-alone noninvasive tool for PCa diagnosis. Hence, this study aims at developing a mpMRI-based radiomic PCa diagnostic model in a cohort of PI-RADS 3 lesions. We enrolled 133 patients with 155 PI-RADS 3 lesions, 84 of which had PCa confirmation by fusion biopsy. Local radiomic features were generated from apparent diffusion coefficient maps, and the four most informative were selected using LASSO, the Wilcoxon rank-sum test (*p* < 0.001), and support vector machines (SVMs). The selected features where augmented and used to train an SVM classifier, externally validated on a holdout subset. Linear and second-order polynomial kernels were exploited, and their predictive performance compared through receiver operating characteristics (ROC)-related metrics. On the test set, the highest performance, equally for both kernels, was specificity = 76%, sensitivity = 78%, positive predictive value = 80%, and negative predictive value = 74%. Our findings substantially improve radiologist interpretation of PI-RADS 3 lesions and let us advance towards an image-driven PCa diagnosis.

## 1. Introduction

Prostate cancer (PCa) is the second most commonly diagnosed cancer in men, with an estimated 1.4 million diagnoses worldwide in 2020 [1], and it expresses a wide clinical variability, from indolent to more aggressive diseases [2]. From a pathological point of view, the 2014 and 2019 International Society of Urological Pathology (ISUP) grading system classifies PCa grades, ranging from 1 to 5 [3], being useful to distinguish clinically significant (csPCa) from not clinically significant (ncsPCa) tumours. In large studies of radical prostatectomy (RP) specimens that showed only ISUP grade 1 disease, in fact, extracapsular extension (0.3%) [4] and biochemical recurrence (3.5%) were rare, and seminal vesicle (SV) invasion or lymph node (LN) metastasis did not occur at all [5,6], noting a very low-risk disease. Conversely, ISUP grade 2 and above, and even ISUP grade 3 and above, are differently considered clinically relevant diseases by many authors, with substantial disagreement on the definition [7,8,9,10]. Nowadays, despite the prostate-specific antigen (PSA) and digital rectal examination remaining essential for the diagnosis of PCa, multiparametric magnetic resonance imaging (mpMRI) and transrectal ultrasound (TRUS) fusion-targeted biopsy significantly improve the detection and localization of ISUP ≥ 2 grade cancers, especially when their diameter is larger than 10mm, with more precise preoperative assessment of both grade and stage [7,8,9,10]. Interpretation and reporting of mpMRI is based on the more recent version 2.1 of the Prostate Imaging Reporting and Data System (PI-RADS), revised in 2019, indicating the probability of csPCa on a five-point scale [11]. In a meta-analysis of 17 studies involving men with suspected or biopsy-proven PCa, the average positive predictive values (PPVs) for ISUP grade > 2 cancers of lesions with a PI-RADSv2.1 score of 3, 4, and 5 were 16% (7–27%), 59% (39–78%), and 85% (73–94%), respectively [12]. Nevertheless, the assessment of PI-RADS scores is still limited by large heterogeneity due to many factors, the most important of which is observer’s experience [13]. This heterogeneity mainly affects the PI-RADS 3 category assessment, which represents an “equivocal” result and can hide a non-negligible probability of csPCa [14]. Proper clinical management of patients with a PI-RADS 3 lesion at the mpMRI represents an intriguing and sometimes distressing challenge for urologists, although it is significantly improved by the use of clinical parameters such as age, familiarity, digital rectal examination (DRE), PSA, PSA density, and recently introduced new tools such as transverse prostate maximum sectional area [15], which can be combined in predictive nomograms. In fact, European Urology Association (EAU) guidelines suggest a risk-adapted matrix table for biopsy management based on the utilization of PSA density (PSAD), which is considered a best predictor of csPCa. For the PI-RADS 3 category, the biopsy can be considered for PSAD 0.10–0.15 ng/mL^2^, and is strongly suggested for PSAD > 0.15 ng/mL^2^ [16]. Recent improvements to identify csPCa besides mpMRI results include the adoption of PSMA-PET to identify suspicious intraprostatic uptake according to PRIMARY score [17], which may be used to target prostate biopsy in equivocal findings at mpMRI. Nevertheless, it would be desirable to improve the categorization of benign vs. malignant lesions in PI-RADS 3 ones in order to reduce the proportion of indeterminate findings. From a radiological point of view, despite the improvements introduced by subsequent versions of PI-RADS, it is now clear that it is not possible to overcome intrinsic limitations of the visual analysis of the images. Radiomics have become popular, as the employment of well-established machine learning and artificial intelligence strategies for analysing radiological images can enrich the information retrieved by visual analysis, even, in fact, beyond that perceivable by the human eye, by means of the generation of quantitative measurements, called radiomic features (RFs), and the employment of the most relevant ones to build a predictive model supporting clinicians’ decisions [18,19,20]. Potentially, using radiomics in PI-RADS 3 lesions evaluation could allow for the diagnosis of benign and malignant lesions by analysing image features only, definitely improving the PCa predictive capability of the PI-RADS 3 score. As a matter of fact, some previous studies have investigated this field [21,22,23,24,25], obtaining promising results. The aim of this study is to develop a machine learning model predictive of PCa in a selected cohort of equivocal PI-RADS score 3 lesions, improving the state of the art through a dedicated automated pipeline of image processing and automatic explainable feature generation.

## 2. Materials and Methods

### 2.1. Study Population

Our local Institutional Review Board approved this observational, retrospective, single-centre study (approval code: 784/2021/Oss/AOUBo) and waived the requirement for informed consent. This study was carried out in accordance with institutional guidelines, including the Declaration of Helsinki. We screened our database of mpMRI exams, conducted according to the PI-RADSv2.1 guidelines at our Radiology Unit from September 2020 to December 2021, in order to collect only PI-RADS 3 lesions. The inclusion criteria were the following: (1) MRI-TRUS fusion targeted biopsy (fusion-TB) only performed at our Radiology Unit; (2) histopathological report from a dedicated genitourinary pathologist of the Pathology Unit of our institution. The exclusion criteria were the following: (1) simultaneous PI-RADS 4 or 5 lesions; (2) mpMRI protocol not strictly adhering to the guidelines’ recommendations; (3) prior surgery for benign prostatic hyperplasia; (4) severe motion or magnetic artifacts altering one or more mpMRI sequences. After the application of inclusion and exclusion criteria, 133 patients with 155 PI-RADS 3 lesions were finally selected and included in our study population.

### 2.2. Image Acquisition

Prostate mpMRI examinations were performed on a 1.5T scanner (Signa HDxt; GE Healthcare, Chicago, IL, USA), using a pelvic phased-array surface coil combined with a disposable endorectal coil. The mpMRI acquisition protocol of the prostate gland and seminal vesicles included fast relaxation fast spin echo (FR-FSE) T2-weighted (T2w), diffusion-weighted imaging (DWI), and dynamic contrast-enhanced (DCE) sequences (scan parameters were reported in our previous study [15]. Apparent diffusion coefficient (ADC) parametric maps were obtained from DWI acquisitions at b = [50, 1000] $\frac{s}{{\mathrm{mm}}^{2}}$.

### 2.3. Biopsy Procedure and Pathological Examination

After receiving antibiotic prophylaxis and a cleansing rectal enema, two highly qualified and expert radiologists (each of them reads ≥250 mpMRI per year and carries out ≥50 mpMRI-TRUS fusion biopsy per year) performed the transrectal biopsies for all PI-RADS 3 lesions by using the mpMRI-TRUS Fusion image guide, a nondisposable biopsy gun (Medgun, Medax, Modena, Italy) with a disposable 18-gauge needle, and a US platform (Canon-Toshiba Aplio 500^TM^, Ōtawara, Kanto, Japan) with an end-fire TRUS probe, as previously described [26,27,28,29]. Two expert genitourinary pathologists examined the tissue specimens, mainly determining whether or not they contained a malignant disease. The ISUP Grade Group System (GGS) was used to assign a score from 1 to 5 to each malignant lesion.

### 2.4. Region of Interest (ROI) Segmentation

The mpMRI studies of included patients were retrieved from our local Picture Archiving and Communication System (PACS). In particular, T2w and ADC series were jointly used for manual segmentation of PI-RADS 3 lesions, whilst ADC only was admitted to quantitative image analysis (i) because of its suitability in evaluating both peripheral and transition zones and (ii) to make the predictive model for PCa diagnosis (hereinafter, PCa diagnostic model) exploitable by biparametric MRI protocols too. Two radiologists with more than ten years of experiences in prostate imaging (C.G. and B.C.) contoured the whole prostate gland and each PI-RADS 3 lesion on ADC maps, checking corresponding findings on T2w. After proper windowing, each radiologist, blinded to biopsy outcome, segmented the regions of interest (ROIs) of patients on which he had previously performed prostate biopsy, on all slices where either prostate or lesions were visible, using ImageJ v1.53 (https://imagej.nih.gov/ij, accessed on 28 September 2022), a Java-based public-domain software.

### 2.5. Machine Learning Pipeline

**Figure 1:**
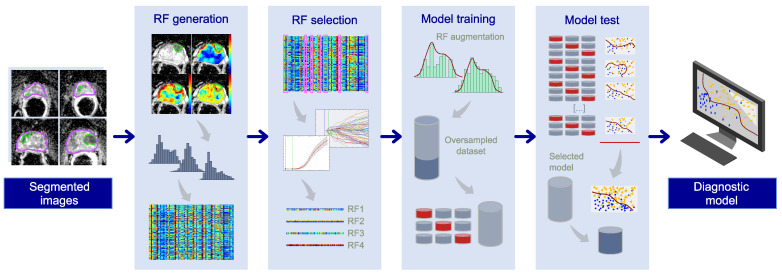
Machine learning pipeline.

The PCa diagnostic model was developed by a bioengineer (M.M.) with 6 years of experience in image processing and data analysis using machine learning methods, implementing a pipeline in MatLab^®^ (R2021b v.9.11, The MathWorks, Natick, MA, USA). Figure 1 illustrates the machine learning pipeline, which takes the segmented images as the input and provides the PCa diagnostic model as the output, throughout the stages described in the following subsections.

### 2.6. Radiomic Feature Generation

Quantitative imaging features, so-called radiomic features (RFs), were generated according to our method proposed in [30], already applied in [31,32,33]. The method is based on a two-stage procedure. First, 12 first-order features (i.e., mean, median, entropy, uniformity, standard deviation, median absolute deviation, interquartile range, coefficient of variation, skewness, kurtosis, mean and median of the last decile of image pixel distribution) were computed locally within each prostate ROI, that is, by assigning each ROI pixel the value of a first-order metric computed in a rectangular window (centred on the pixel itself). In particular, a window of 9 × 9 pixel size was adopted, based on a previous report [33]. This stage provided us with 12 stacks of parametric maps for each patient, displayed as hot–cold colorimetric maps, and as many local first-order features, for all prostate and lesion slices. Then, the feature values of the PI-RADS 3 lesion ROIs in all slices were extracted from first-order parametric maps and their global distributions (i.e., of all slices) were described through the same 12 first-order features as above. After that, the second stage provided us with 144 RFs. At the end, the 12 first-order features were also derived from ADC maps within lesion ROIs; altogether, this yielded 156 RFs for each PI-RADS 3 lesion.

### 2.7. Radiomic Feature Selection

On the basis of the sample size of the smallest class of the dataset (i.e., 71 PI-RADS 3 benign lesions), a selection procedure was performed to derive the most informative combination (i.e., a radiomic signature) from the entire set of 156 generated RFs. After preliminary tests, a combination was chosen of four RFs at most, thus minimizing the risk of overfitting. The procedure for feature selection consisted of two main stages. First, after RF standardization, a preliminary subset of the most informative RFs arose from the least absolute shrinkage and selection operator (LASSO) regression, employing 10-fold cross-validation (CV) at the minimum CV error rule, and weighing each sample by its prior probability. Second, the most informative combination of four RFs was derived from the LASSO subset, by performing a preliminary discriminatory study. In particular, the discriminatory capability of all possible combinations of 4 different RFs was assessed through as many support vector machines (SVMs). Hence, their discriminatory performance was ranked by the *p*-value of the Wilcoxon rank-sum test (*p* < 10${}^{-3}$), corrected by Holm–Bonferroni, and in case of equivalent *p*-values, by the maximum Youden index (Y.I.) of the corresponding receiver operating characteristic (ROC) curve of each SVM-based diagnostic test, which is equal to specificity(SP)+sensitivity(SN)-1. In view of the subsequent training phase of the PCa diagnostic model, this procedure was repeated twice, by using 1st- and 2nd-order polynomial SVM kernels, respectively, yielding two radiomic signatures employed for developing as many PCa diagnostic models.

### 2.8. Training of the Prostate Cancer (PCa) Diagnostic Model

Once selected, the RFs were admitted to data augmentation, exploiting the procedure described in [32], in order to strengthen the statistical significance of the data subsets used for training, validation, and test phases. By augmenting the initial dataset by 60%, the resulting oversampled dataset (OD) was constituted by 248 samples, split between 114 PIRADS 3 benign lesions and 134 PIRADS 3 PCa. To build the PCa diagnostic model, we exploited an SVM classifier with both linear and 2nd-order polynomial kernels, to compare their predictive performance. In particular, the SVM was chosen for its suitability for working at best even on small datasets. In fact, the placement of the SVM hyperplane in the feature space is given by just the coordinates of the support vectors (SVs), thus contributing to minimize the risk of overfitting. The entire OD was split between training (70%) and holdout test (30%) subsets according to the method used in [27,30,32], exploiting the SVM margin rule to ensure the statistical representativeness of subsets. For model training, the PIRADS 3 lesions being diagnosed as PCa were the positive class, whilst the benign lesions were the negative one. On the training set, 100 runs of 3-fold CV were performed, with each CV fold having 31 positive samples and 27 negative ones. For each SVM run, the hyperparameter tuning was performed using the MatLab Bayesian optimization algorithm. Finally, the training phase yielded 300 competing models.

### 2.9. Final Model Selection and Holdout Test Phase

The last model selection phase has to detect and tune the ultimate PCa diagnostic model, to be tested on the holdout subset. The procedure consisted of two main stages. First, among each run, which yielded three SVM classifier models (stemming from 3-fold CV), one only was kept, that is, that leading to the highest Area Under the ROC (AUC) on the test fold, after discarding models prone to overfitting, where AUC on the training folds was lower than on the test fold. This stage reduced the number of competing models to 100. Then, to increase the robustness of the final PCa diagnostic model, all 100-sample distributions of the SVM kernel parameters were considered. Hence, the median value of each distribution was calculated and used to train the final optimized model. Accordingly, a separating hyperplane was defined for each SVM kernel, and its corresponding ROC curve referred to the entire training subset was assessed. As regards the SVM bias term, it was adjusted to meet the radiomic score at the Y.I. of the ROC. The PCa diagnostic models (arising from the linear and the 2nd-order polynomial kernels) were tested on the holdout subset, and model performance was measured through SN, SP, I, positive predictive value (PPV), and negative predictive value (NPV). In addition, the performance was visually assessed through boxplots and waterfall plots. Finally, the radiomic score on the holdout test subset was converted into posterior probability using a binomial logit function.

### 2.10. Literature Search

Our diagnostic performance was compared with peer-reviewed articles published on international journals, identified by querying PubMed and Google Scholar database with ((radiomic AND “PI-RADS 3”) AND (radiomic AND “PIRADS 3”)) regular expressions, and filtering results since 2019, to select studies compliant with PI-RADS v2.1 definition.

## 3. Results

### 3.1. Patient Characteristics

Patient and lesion characteristics are summarized in Table 1.

### 3.2. Radiomic Signature

Of the entire set of generated RFs (i.e., 155), the 13 most relevant RFs were selected by LASSO, this yielding 715 possible combinations of four different RFs, fed to the second stage of feature selection. The most discriminant combination (with *p* ∼ 10^−10^), arising from the discriminatory study performed by linear SVM, consisted of the standard deviation computed on the parametric map of the local median (M–*σ*, RF55), the mean computed on the map of the mean value of the last decile (*μ*_90th_–*μ*, RF61), the entropy of the map of the median value of the last decile (M_90th_–e, RF83), and the *σ* of the skewness map (S–*σ*, RF91). Instead, the discriminatory study performed by polynomial SVM yielded 13 combinations, having *p* ∼ 10^−11^, where the best one, with the highest I value (I = 0.57), was constituted by M–*σ*, *μ*_90th_–*μ*, S–*σ* (as for the linear SVM study), and the skewness was computed on the parametric map of the local mean (*μ*–S, F39). Table 2 resumes the meaning of the five different selected RFs, by indicating, separately, the local parametric map they refer to and the global descriptor computed upon.

### 3.3. PCa Diagnosis among PI-RADS 3 Lesions

**Figure 2:**
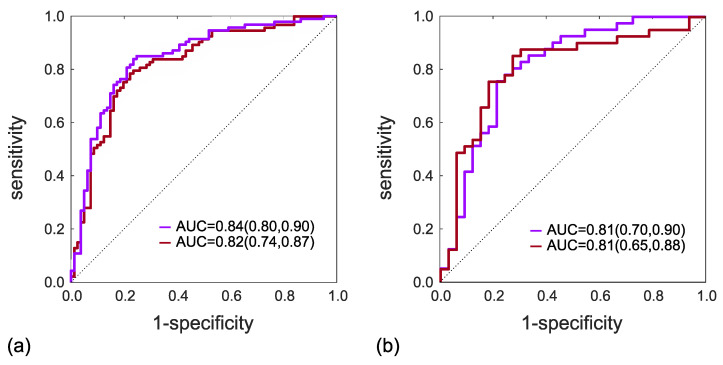
ROC curves of the PCa diagnostic models of training (**a**) and test (**b**) subsets, using both linear (red line) and 2nd-order polynomial (purple line) SVM kernels.

Figure 2 reports the ROC curves of the PCa diagnostic models referred to training (a) and holdout test (b) subsets, using both linear (red line) and second-order polynomial (purple line) SVM kernels, whose AUC values, in training, are equal to 0.82 (95% C.I. 0.74, 0.87) for linear and 0.84 (95% C.I. 0.80, 0.90) for polynomial kernels, whilst in test, AUC values are 0.81 (95% C.I. 0.70–0.90) for linear and 0.81 (95% C.I. 0.65, 0.88) for polynomial kernels.

**Figure 3:**
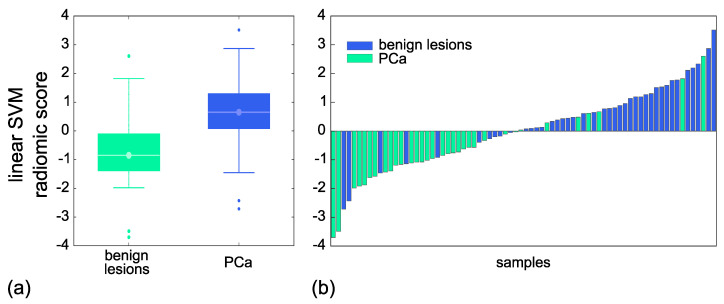
Boxplot (**a**) and the waterfall plot (**b**) of the linear SVM radiomic score referred to the test set.

By referring to the Y.I. of the ROC curves, the diagnostic model developed by the linear SVM predicted PCa, in training, with 18 FP and 20 FN, this corresponding to SP = 78% and SN = 78% (Y.I. = 0.56). Instead, in test, PCa diagnosis was achieved with 7 FP and 10 FN, which yielded SP = 79% and SN = 76% (Y.I. = 0.54). Then, Figure 3 also reports the boxplot (a) and the waterfall plot (b) of the linear SVM radiomic score referred to the test set.

**Figure 4:**
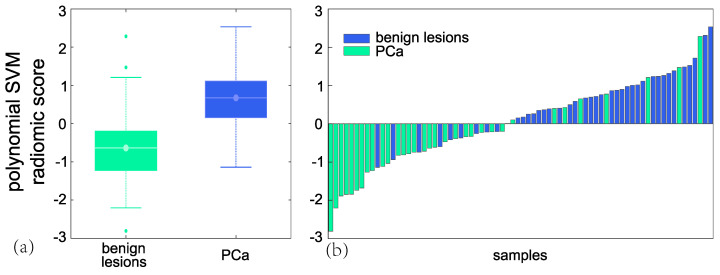
Boxplot (**a**) and the waterfall plot (**b**) of the 2nd-order polynomial SVM radiomic score referred to the test set.

As regards the polynomial SVM model, the prediction of PCa lesions was achieved, in training, with 19 FP and 15 FN, SP = 77% and SN = 84% (Y.I. = 0.60), whilst in test with 8 FP and 9 FN, SP = 76% and SN = 78% (Y.I. = 0.54). Figure 4 reports the boxplot (a) and the waterfall plot (b) of the second-order polynomial SVM radiomic score referred to the test set. As regards the clinical performance of the diagnostic model in the test subset, PPV and NPV values are 82% and 72%, respectively, by linear SVM, and 80% and 74%, respectively, by polynomial SVM.

Finally, Table 3 reports the performance of both diagnostic models referred to the training and test subsets.

### 3.4. Literature Analysis

A total of 81 scientific articles resulted from the Google Scholar query and 29 from the PubMed, one, all included in Google Scholar results. Of these, 4 studies focussed on PCa diagnosis among selected cohorts of equivocal PI-RADS 3 lesions [21,22,23,24,25]. Table 4 reports the performance of these previous studies in terms of enrolled patients and the number of PI-RADS 3 lesions, the number of RFs included in the final model, AUC, SP, SN, and Y.I. The table also reports if the model was tested on a holdout or external subset.

## 4. Discussion

Category 3 of the PI-RADS score, indicating an equivocal likelihood of csPCa, represents an intriguing clinical challenge between avoiding unnecessary biopsies and improving PCa detection. In our study population, 61% of patients with at least one PI-RADS 3 lesion at mpMRI underwent fusion targeted biopsy despite the PSA density being ≤ 0.15 ng/mL^2^ due to several reasons, such as other suspicious clinical data (including DRE and familiarity) or an evaluation before the last change of EAU guidelines. This trend still manifests a difficulty in the management of PI-RADS 3 lesions in daily clinical practice, which can affect the complete adherence to the guidelines. Moreover, 36% of PI-RADS 3 lesions in our study population resulted in PCas with GGS ≥ 2, of which 14% of GGS ≥ 3, suggesting that some important limitations still remain in the adequate radiological assessment, and that some efforts can be made to improve the correct categorization of benign vs. malignant lesions visible on mpMRI. Starting from the optimistic results offered by radiomic analysis in the PCa detection and evaluation compared to the PI-RADS assignment, we investigated the capability of the radiomic approach of improving the stratification of lesions in the PI-RADS 3 category, which is potentially useful in the future to determine a downgrade to PI-RADS 2 or an upgrade to PI-RADS 4 for some lesions and to reduce the proportion of indeterminate lesions.

Therefore, using biopsy as the reference standard, we developed two diagnostic models for PCa, based on ADC radiomics, adopting SVM classifiers with two kernel types, linear and second-order polynomial, each based on a combination of four RFs only. In particular, three out of four RFs were shared between the two models (i.e., M–*σ*, *μ*_90th_–*μ*, and S–*σ*), this strengthening the informative role held by the selected RF vectors. Both models could achieve high performance in diagnosing PCa among PI-RADS 3 score lesions, with PPV values always higher than 80% while NPV was kept not lower than 72%, this enforcing the stand-alone role of mpMRI imaging (and ADC series) endowed with machine learning tools, as a noninvasive alternative to better select patients with PI-RADS 3 who really need prostate biopsy. The potential value of our findings is even higher if we consider the limited number of exploited RFs with respect to the sample population, which increases the generalizability of the diagnostic model on wider populations. In particular, as regards diagnostic performance, the polynomial model shows similar training performance to the linear one, and equivalent performance in the holdout test subsets, with the same AUC and number of total errors (i.e., 17) that yield the same Y.I. values (i.e., Y.I. = 0.54). Nevertheless, the polynomial model should be preferred in order to have a balanced distribution of FP and FN errors, which yields more similar values of SP and SN, and of PPV and NPV values, accordingly. Our findings also confirm the well-established role of ADC radiomics, already exploited in a previous study [33], and highlight the feasibility of single-sequence radiomics, thus also minimizing, in the view of a prospective implementation of radiomic tools in the clinical practice, the computing time, favouring a reduction in patient motion, and the time required to clinicians for image series segmentation, after acquisition.

Even considering studies from the literature, ADC radiomics is always explored [21,22,23,24,25], and most of the time, it is included in the predictive model showing the highest performance [21,23]. Actually, not all studies from the literature comparison (Table 3) report sufficient metrics to allow for an exhaustive comparison to our performance. For instance, two studies [21,22,23,24,25] report exclusively AUC values. As regards the study in [20], not any validation, either internal or external, is reported, so the AUC value should be compared to that achieved in the training subset, which is somewhat higher. Moreover, results in [21] are achieved with a notably high number of RFs with respect to their sample size. Instead, the study in [23] reports substantially a lower value of AUC. Similarly, the study in [24] shows worse performance than ours when considering, besides AUC, the Y.I., a summarizing index of SP and SN metrics, which is much lower. The study in [22] is the only one showing higher performance than ours, being driven by an incredibly high number of RFs, even greater than the total sample size, thus substantially depicting an overfitted model.

Moreover, the achieved results represent a marked step forward in confirming the capability of radiomics to improve the discrimination between benign and malignant lesions, by integrating the evaluation through the PI-RADS score and mainly using the diffusion sequence. This could confirm the efficacy of the biparametric versus multiparametric MRI approach, with a consequent reduction in costs and execution times.

In conclusion, our study represents a proof-of-concept of the determinant contributions stemming from the ML analysis of mpMRI images for improving the diagnostic capability of radiological images, with early and noninvasive tools. Notably, the benefits of ML analysis have been proved even on the diagnosis of PCa in a selected cohort of PI-RADS 3 lesions, whose clinical management is still uncertain. Differently from what was reported in the reviewed literature, the methodology adopted bounded the possibility of overoptimistic predictive performance, thus allowing us to be confident on the robustness of results, although this needs to be confirmed on a wider population.

Actually, the present study also has some limitations. First of all, this study was a retrospective analysis of a relatively small group of patients from a single institution, with a single scanner, and before its wider clinical application, our predictive model needs to be prospectively validated on a larger scale, with patients from other medical units using different MRI scanners. Second, a separate analysis of PZ and TZ lesions was not performed due to too insufficient a sample size, and this will be pursued on a wider population. Finally, we used biopsy as the reference standard, which can be affected by undersampling errors; thus, a correlation with surgical specimens can be more appropriate, although from a methodological point of view, this implies, at most, a negative bias on the performance of the model. A future development, requiring a larger cohort, could be represented by the integration of radiomic analysis and clinical data, such as PSAD, in order to improve the accuracy of the model predictive of PCa and enforce a tailored approach in selecting patients with PI-RADS 3 lesions who need a biopsy.

## Figures and Tables

**Table 1:** Characteristics of study population.

Parameter		Value
**Patients (n°)**		133
Age		
	μ±σ, years	69 ± 6
	range, years	51–88
PSA		
	μ, ng/mL^2^	8
	range, ng/mL^2^	1.59–46
PSAD		
	<0.15, ng/mL^2^	81
	≥0.15, ng/mL^2^	52
**PI-RADS 3 lesions (n°)**		155
Size		
	median, mm^2^	119
	IQR, mm^2^	177
PZ		115
TZ		40
Negative biopsy (n°)		71/155 (46%)
ISUP 1 (n°)		28/84 (33%)
	PSAD < 0.15, ng/mL^2^	14
	PSAD ≥ 0.15, ng/mL^2^	14
ISUP 2 (n°)		34/84 (40%)
	PSAD < 0.15, ng/mL^2^	16
	PSAD ≥ 0.15, ng/mL^2^	18
ISUP 3 (n°)		10/84 (12%)
	PSAD < 0.15, ng/mL^2^	8
	PSAD ≥ 0.15, ng/mL^2^	2
ISUP 4 (n°)		8/84 (10%)
	PSAD < 0.15, ng/mL^2^	2
	PSAD ≥ 0.15, ng/mL^2^	6
ISUP 5 (n°)		4/84 (5%)
	PSAD < 0.15, ng/mL^2^	1
	PSAD ≥ 0.15, ng/mL^2^	3

**Table 2:** Selected radiomic features.

Radiomic Feature (RF)		Local Parametric Map	Global Descriptors
Number	Identifier		
RF39	μ–S	mean (μ)	skewness (S)
RF55	M–*σ*	median (M)	standard deviation (*σ*)
RF61	*μ*_90th_–*μ*	*μ* of the last decile (*μ*_90th_)	*μ*
RF83	M_90th_–e	M of the last decile (M_90th_)	entropy (e)
RF91	S–*σ*	mean (*μ*)	S

**Table 3:** Performance of diagnostic models.

RFs	SVM Kernel	Subset	AUC	FP/P	FN/N	SP	SN	Y.I.	PPV	NPV
RF[55-61-83-91]	Linear	Training	0.82	18/93	20/81	78%	78%	0.56	80%	76%
		Test	0.81	7/41	10/33	79%	76%	0.54	82%	72%
RF[39-55-61-91]	2nd-order	Training	0.84	19/93	15/81	77%	84%	0.60	80%	81%
	polynomial	Test	0.81	8/41	9/33	76%	78%	0.54	80%	74%

**Table 4:** Literature analysis.

Study	Series	Pz	PI-RADS 3	PCa *	RFs	Test	AUC	SP (%)	SN (%)	Y.I.
2020, Li et al. ^2^ [22]	mpMRI	36	36	6	45	yes	0.94	100	80	0.80
2021, Giambelluca et al. ^1^ [21]	ADC	43	46	19	6	no	0.82	–	–	–
2021, Lim et al. ^2^ [23]	T2w, ADC	158	160	80	10	yes	0.68	–	–	–
2021, Brancato et al. ^1,2^ [24]	T2w	–	41	26	2	yes	0.76	51	80	0.31
2023, Jin et al. ^1,2^ [25]	–	463	80 ^3^	26 ^3^	3	yes	0.75	72	85	0.57
**our work** ^2^	**ADC**	133	74 ^†^	41	4	yes	0.81	76	78	0.54

^1^ Reported data refer to the predictive model having the highest performance among those presented by the authors. ^2^ Reported results refer to the test subset. ^3^ Reported data refer to the external validation set. * Number of non-PCa lesions can be derived by subtracting data in “PCa” from data in “PI-RADS 3” columns. ^†^ This number refers to augmented lesions within the test subset.

## Data Availability

The data are not available because of patient privacy.
